# Primary cilia TRP channel regulates hippocampal excitability

**DOI:** 10.1073/pnas.2219686120

**Published:** 2023-05-22

**Authors:** Thuy N. Vien, My C. Ta, Louise F. Kimura, Tuncer Onay, Paul G. DeCaen

**Affiliations:** ^a^Department of Pharmacology, Feinberg School of Medicine, Northwestern University, Chicago, IL 60611; ^b^Center for Genetic Medicine, Feinberg School of Medicine, Northwestern University, Chicago, IL 60911

**Keywords:** polycystins, TRP channels, primary cilia, channelopathy, ciliopathy

## Abstract

Polycystins (PKD2, PKD2L1, and PKD2L2) are members of the transient receptor potential family, which form ciliary ion channels. Most notably, PKD2 dysregulation in the kidney nephron cilia is associated with polycystic kidney disease, but the function of PKD2L1 in neurons is undefined. In this report, we develop animal models to track the expression and subcellular localization of PKD2L1 in the brain. We discover that PKD2L1 localizes and functions as a Ca^2+^ channel in the primary cilia of hippocampal neurons that apically radiate from the soma. Loss of PKD2L1 expression ablates primary ciliary maturation and attenuates neuronal high-frequency excitability, which precipitates seizure susceptibility and autism spectrum disorder–like behavior in mice. The disproportionate impairment of interneuron excitability suggests that circuit disinhibition underlies the neurophenotypic features of these mice. Our results identify PKD2L1 channels as regulators of hippocampal excitability and the neuronal primary cilia as organelle mediators of brain electrical signaling.

## Significance

Primary cilia are antenna-like organelles that represent a frontier of knowledge in neuroscience research. They were first reported by Duncan and Dahl more than 60 years ago, and although they are implicated in neurodevelopmental diseases, our understanding of their function in neurons is limited. In this manuscript, the authors demonstrate that the primary cilium is an excitable organelle richly populated with PKD2L1 ion channels. Using microelectrode electrophysiology and mouse genetics, the authors find that these channels in the cilia contribute to high-frequency action potential firing and their loss of function primarily impacts interneuron excitability. Loss of PKD2L1 expression impairs ciliary maturation in mice, which behaviorally exhibits autism-like features and seizure susceptibility that may have implications to human neuronal ciliopathy conditions.

The primary cilium is a privileged, microtubule-based organelle found on the apical side of polarized cells. Various neurons of the central nervous system (CNS) produce a single primary cilium that extend from the basal body into the interstitial space (Dahl 1;, Duncan 2;). Primary cilia are proposed chemosensory antennae in the CNS—receiving neuromodulatory inputs through activation of enriched G-protein-coupled receptors (3–6). The so-called ciliopathies are genetic disorders caused by variant proteins related to ciliary function (7). Features of ciliopathies range from embryonic lethality to conditions that impact specific organs, such as autosomal-dominant polycystic kidney disease (ADPKD) and neurodevelopmental disorders such as Meckel–Gruber syndrome (8, 9). However, the mechanistic dysregulation of primary ciliary signaling underlying most ciliopathies is poorly understood. Neuronal ciliopathies are associated with autism spectrum disorder (ASD) and epilepsy (10–12). ASD is a prevalent group of neurodevelopmental psychiatric disorders characterized by deficits in social interactions, interpersonal communication, and repetitive and stereotyped behaviors (13). Epilepsy is a disorder of the brain characterized by repeated seizures caused by focal and widespread electrical discharges. Excitatory and inhibitory imbalances of neuronal circuits are considered key mechanistic features of the pathophysiology underlying ASD and epilepsy, the genetic causes of which are often associated with ion channel variants (14). While the significance of primary ciliary contributions to cortical development is mounting, their functional regulation of neuronal circuitry and neuronal excitability is largely undefined (3).

Polycystin genes (PKD2, PKD2L1, and PKD2L2) encode a subclass of the transient receptor potential (TRP) family of ion channels (15–17). Polycystins are also referred to by their TRP channel nomenclature (TRPP1, TRPP2, and TRPP3), but the revised nomenclature creates ambiguity regarding their genetic identity (18). For simplicity, we will refer to the polycystin proteins by their gene name rather than differentiating gene and protein with separate names, as proposed in a recent review (19). Each polycystin subunit has 6 transmembrane helices that homotetramerize to form a voltage-dependent cation conductance (20–23). Polycystins can also heterooligomerize with PKD1-related channel-receptor subunits (PKD1, PKD1L1, PKD1L2, PKD1L3, and PKDREJ), several of which are proposed to functionalize as a ligand-dependent or autoproteolytically activated channel complexes (24–26). PKD2 and PKD2L1 traffic to primary cilia of disparate tissues—a unique feature of this TRP channel subfamily (17, 27, 28). Variants in PKD2 dysregulate the channel’s function in the primary cilia of kidney collecting duct cells and are associated with ADPKD; a renal ciliopathy and a common monogenic disorder (9, 29, 30). Unlike PKD2, variants in PKD2L1 have yet to be linked with human disease. Nonetheless, phenotypes of murine knockout animals implicate this channel’s role in sour-taste reception, laterality defects, and hippocampal–thalamocortical activity (31–33). PKD2L1 is expressed across several regions of the fetal and adult brain (34, 35). Herein, we explore PKD2L1’s role in the hippocampus by genetically labeling the channel and tracking its subcellular distribution to the primary cilia of neurons. We directly recorded PKD2L1 activity from the primary ciliary membrane and identified its function supporting high-frequency neuronal excitability. Loss of PKD2L1 function impairs primary ciliary maturation and manifests in ASD-like features with enhanced seizure susceptibility in mice—implicating the channel and primary ciliary organelle as regulators of brain electrical signaling.

## Results

**Fig. 1.**
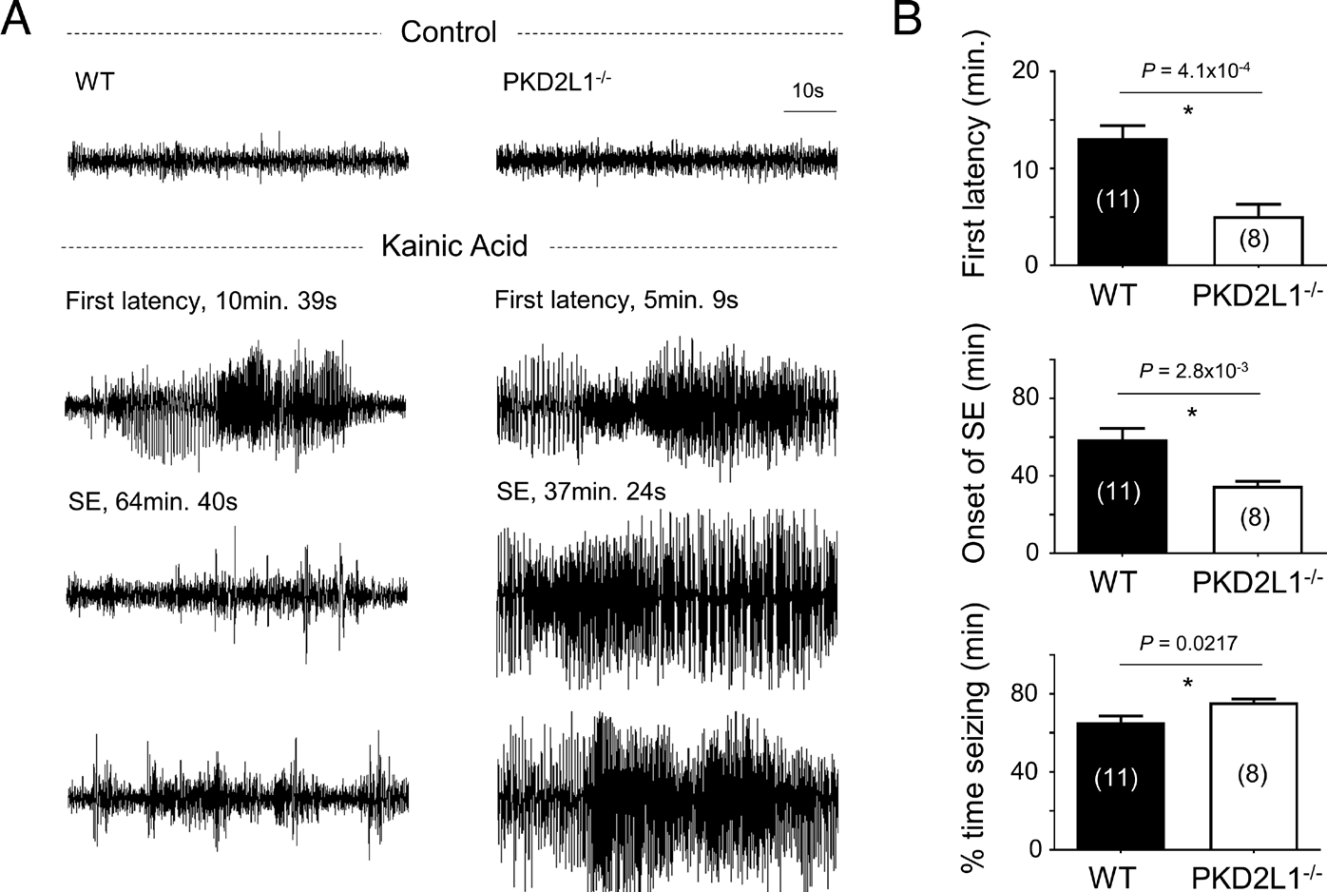
Loss of PKD2L1 causes enhanced seizure susceptibility in mice. (*A*) Examples of 1 min EEG traces in mice at baseline before (control) and after kainic acid injection (kainic acid). Top row of kainic acid traces indicates 1 min results from WT (*Left*) and PKD2L1^−/−^ (*Right*) mice during the first epileptiform activity following kainic acid. Middle and bottom traces are 1 min results during the epileptiform activity observed at the onset of SE in WT (*Left*) and PKD2L1^−/−^ (*Right*) mice. (*B*) The time to first epileptiform activity (first latency, *Top*), onset of SE (onset of SE, *Middle*), and total percentage of time spent seizing (% time seizing, *Bottom*) were measured for WT and PKD2L1^−/−^ mice. Sample sizes are indicated within parenthesis. Error bars are equal to SD. *P* values from paired Student’s *t* tests are indicated above the graphs. Asterisk indicates significant *P* values < 0.05.

Previous work reported that complete loss of PKD2L1 expression in mice (PKD2L1^−/−^) caused enhanced excitability of cranial local field potentials and susceptibility to pentylenetetrazol-induced seizures (31). However, the proposed function of PKD2L1 within CNS neurons is uncharacterized, as is the mechanism by which loss of this channel manifests in the seizure phenotype. We began our study by assessing PKD2L1’s allelic contribution through a complete characterization of the neurophenotype of hypomorphic PKD2L1 mice. We assessed heterozygous (PKD2L1^+/−^) and homozygous (PKD2L1^−/−^) knockout mouse sensitivity to seizure induction using a kainic acid challenge while monitoring brain electrical activity using implanted cranial electroencephalography (EEG) electrodes. Consistent with previous results (31), we find that PKD2L1^−/−^ mice are significantly more prone to seizure events with faster first latency, total seizing time, and onset of status epilepticus (SE) (Fig. 1 *A* and *B*). As discussed in the introduction, seizure disorders are frequently reported as medical comorbidities in individuals with ASD. To further characterize the neuronal phenotype, we compared wild type (WT) and PKD2L1^−/−^ mice in a series of behavioral tests (36) commonly used to identify ASD-like features in rodents (*SI Appendix*, Fig. S1 *A*–*D*). PKD2L1^−/−^ mice exhibit significant increased repetitive/stereotyped motor behaviors (marble burying), deficits in locomotor activity (open field), enhanced behavioral anxiety (zero maze), and reduced social interaction (three-chamber). Similar, but attenuated, differences were observed in heterozygous PKD2L1^+/−^ mice, suggesting a gene–dose effect on this phenotype (*SI Appendix*, Fig. S1 *A* and *B*). No differences were observed in the Y-maze results comparing WT and PKD2L1^−/−^ mice, suggesting that no deficits in spatial memory are associated with loss of PKD2L1 expression (*SI Appendix*, Fig. S1*E*).

**Fig. 2.**
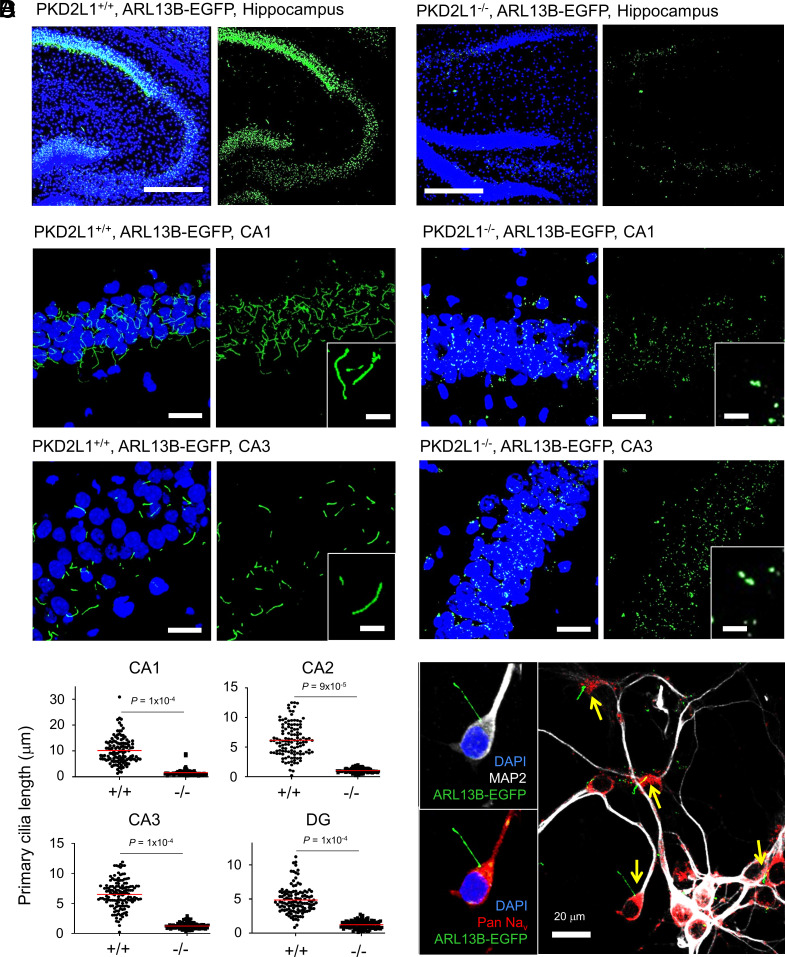
Loss of PKD2L1 expression impairs primary ciliary maturation of hippocampal neurons. (*A*) Global stitched confocal images of sectioned hippocampal slices visualizing primary cilia (transgene ARL13B-EGFP, green) and nuclei labeled with DAPI (staining, blue). (*B*) Confocal images of the CA1 and CA3 regions of the hippocampus. *Inset*, expanded views of mature and immature primary cilia taken from PKD2L1^+/+^ or PKD2L1^−/−^ mice. (*C*) Analysis of the primary ciliary length from the CA1 (N = 120 for each genotype), CA2 (N = 120 for each genotype), CA3 (N = 140 for each genotype), and dentate gyrus (N = 120 for each genotype). *P* values from paired *t* tests for each region of the hippocampus are indicated above the graphs. Average ciliary length is indicated by red lines. (*D*) Confocal images of fixed cultured PKD2L1^+/+^ neonatal hippocampal neurons labeled with antibodies for microtubule-associated protein-2 (MAP2) and pan-subtype voltage-gated sodium channels (Pan-Na_v_). Location of primary cilia (ARL13B-EGFP) sprouting from the soma (DAPI) is indicated by yellow arrows.

Since PKD2L1 is a known primary ciliary channel subunit (28) and since the murine knockout phenotype implicates deficiencies in hippocampal function, we hypothesized that PKD2L1 may alter neuronal ciliary stability in this brain region. Using confocal microscopy of brain sections harvested from mice expressing a fluorescent ciliary reporter (ARL13B-EGFP), we observed two striking anatomical features (32). First, hippocampal neurons within the CA1–CA3 molecular layer exhibit radially oriented primary cilia, with cilia from the CA1 (average 8.9 µm) being the longest (Fig. 2 *A–C* and Movie S1). Second, hippocampal cilia from this layer sprout from the soma, a compartment that does not initiate action potentials, rather contributes to spiking and supports their backpropagation from the axon’s initial segment to dendrites (Fig. 2*D*) (37). However, when brain slices were imaged from ARL13B-EGFP mice backcrossed to the PKD2L1^−/−^ strain (PKD2L1^−/−^:ARL13B-EGFP), the primary cilia from the *cornu Ammonis* 1 to 3 (CA1 to CA3) and dentate gyrus (DG) failed to mature—remaining submicron in length and colocalized with immunolabeled γ-tubulin of the centriole from which they originate (Fig. 2 *A*–*C* and *SI Appendix*, Fig. S2*A*, and Movie S1). The lack of primary cilia from this brain region was confirmed by immunolabeling for the ciliary protein, acetylated tubulin (*SI Appendix*, Fig. S2*B*). The immature ciliary phenotype was also observed in neurons of the cortex (*SI Appendix*, Fig. S2*C*). However, when we examined cilia from tissues outside of the CNS—including the kidney collecting duct, liver, spleen, and photoreceptors of the retina—their length was not affected by the loss of PKD2L1 expression (*SI Appendix*, Fig. S3 *A*–*D*). These results indicate that defects in ciliary morphology caused by ablating PKD2L1 are not systemic and possibly restricted to cells of neuronal lineage.

**Fig. 3.**
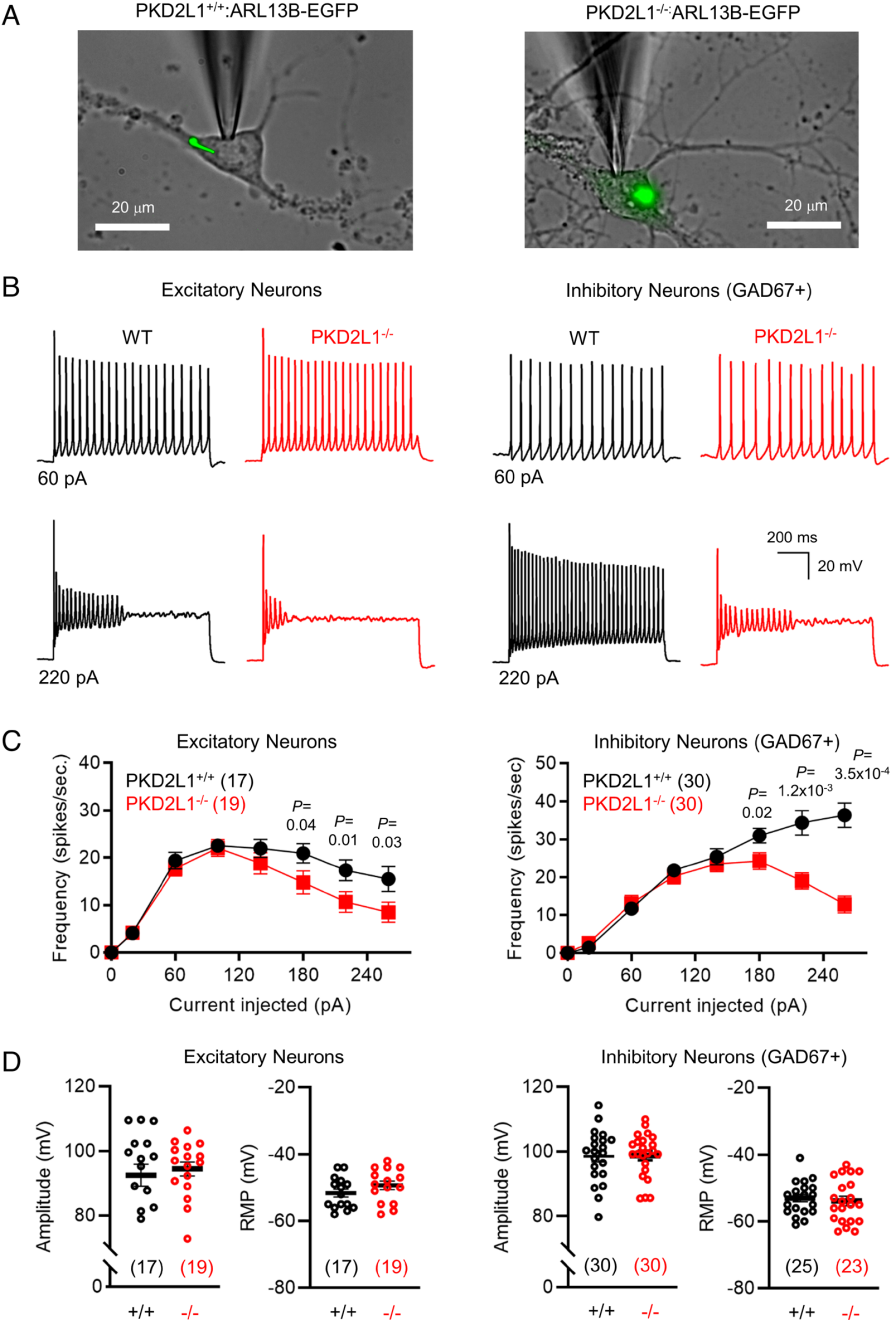
PKD2L1 channel expression contributes to hippocampal neuron excitability. (*A*) Images of current-clamped hippocampal neurons isolated from PKD2L1^+/+^: ARL13B-EGFP and PKD2L1^−/−^: ARL13B-EGFP mice. (*B*) Example action potential recordings taken from excitatory and inhibitory neurons of both genotypes after 8 to 14 d in culture. (*C*) Average spike frequency–current relationships recorded from excitatory and inhibitory neurons. *P*-values (*P *< 0.05) resulting from paired Student’s *t* tests are indicated. (*D*) Maximal spike amplitude and resting membrane potential (RMP) of excitatory and inhibitory neurons. Averages are represented by horizontal black line. The number of neurons in each sample size is indicated in the parenthesis. Error bars are equal to SEM.

The neuro-ciliary phenotype of PKD2L1^−/−^ mice suggests the channel’s and organelle’s role in regulating CNS excitability. However, the impact of PKD2L1 expression on neuronal action potential firing is untested. To evaluate this, we conducted current clamp recordings from the soma membrane of dissociated neonatal hippocampal neurons with primary cilia illuminated with the ARL13B-EGFP fluorophore (Fig. 3*A*). Patch electrodes were loaded with biocytin to identify the recorded neuron under confocal microscopy post hoc (*SI Appendix*, Fig. S4). The data were divided into excitatory and inhibitory neuron populations based on morphology and immunoreactivity after fixation. Excitatory pyramidal-shaped cells were identified by their bulbous, triangular cell bodies with prominent proximal dendritic extensions, whereas inhibitory interneurons were identified by bipolar processes and intense soma-localized glutamic acid decarboxylase (GAD+) immunolabeling (*SI Appendix*, Fig. S4). Expression of PKD2L1 had no impact on the resting membrane potential, action potential amplitude, and spike frequency under low-current (≤ 140 pA) injection (Fig. 3 *B–D*). However, spike frequency at high-current injection (≥160 pA) from neurons isolated from PKD2L1^−/−^ mice was significantly lower for both excitatory and inhibitory neurons, with the latter being more profoundly attenuated (Fig. 3 *B* and *C*). These results suggest that PKD2L1 channel activity influences high spike frequency electrical transmission and implicates disinhibition via impairment of interneuron excitability as a mechanism that underlies the epilepsy–ASD phenotype PKD2L1^−/−^ mice.

**Fig. 4.**
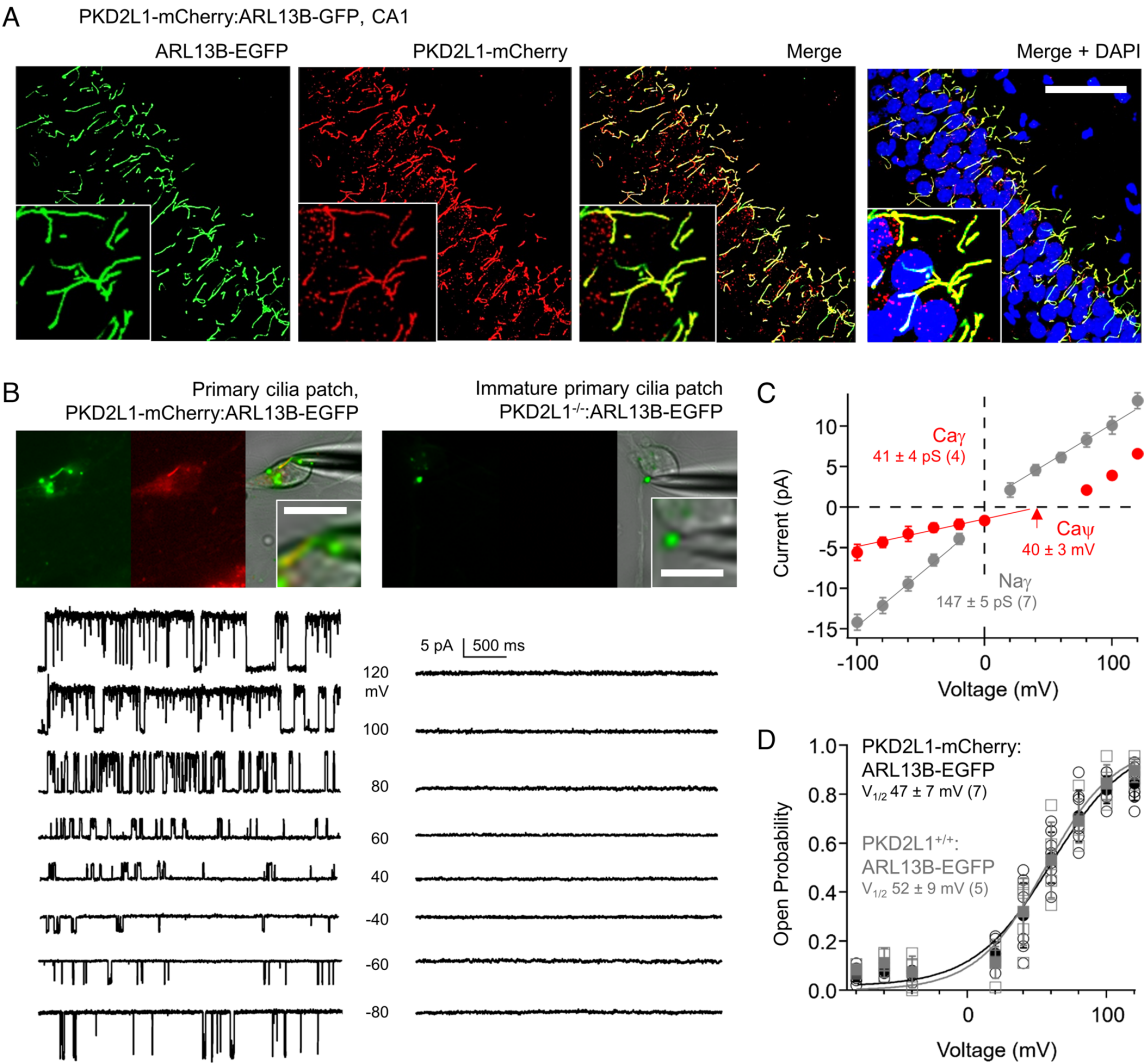
PKD2L1 forms an ion channel in hippocampal neuron primary cilia. (*A*) Hippocampal section histology from the CA1 demonstrating the colocalization of PKD2L1-mCherry (red) into the primary ciliary compartment illuminated by ARL13B-EGFP (green) of neurons. (Scale bar, 20 µm.) (*B*) *Top*, images of voltage-clamped mature primary cilia (PKD2L1-mCherry) and immature (PKD2L1^−/−^) primary cilia from cultured hippocampal neurons. (Scale bar = 5 µm.) *Left*, example single-channel currents recorded from the cilia of PKD2L1-mCherry:ARL13B-EGFP neurons using 140 mM NaCl within the patch pipette. Open-channel events were detected in 7/9 ciliary patches using Na^+^ as the external charge carrier and from 4/5 patches using Ca^2+^ as a charge carrier. *Right*, current records from immature neuronal cilia isolated from PKD2L1^−/−^:ARL13B-EGFP mice using the same saline Na^+^ conditions. No open-channel events were detected from immature ciliary patches (N = 15, total), where N = 12 high-resistance seals were made using Na^+^ as an external charge carrier and N = 3 using Ca^2+^. (*C*) Average single-channel current amplitude–voltage relationships measured from PKD2L1-mCherry:ARL13B-EGFP neuronal primary cilia. Inward single-channel conductances (γ) were estimated from a linear fit of the data generated from experiments using either Na^+^ or Ca^2+^ within the recording electrode. (*D*) Single-channel current open probability (Po)–voltage relationships measured from PKD2L1-mCherry:ARL13B-EGFP (black) and PKD2L1^+/+^:ARL13B-EGFP (gray) neuronal primary cilia. Half-maximal open channels (V_1/2_) were estimated by fitting the data to a Boltzmann equation. In each graph, error bars indicate SD and sample sizes are indicated within parenthesis.

**Fig. 5.**
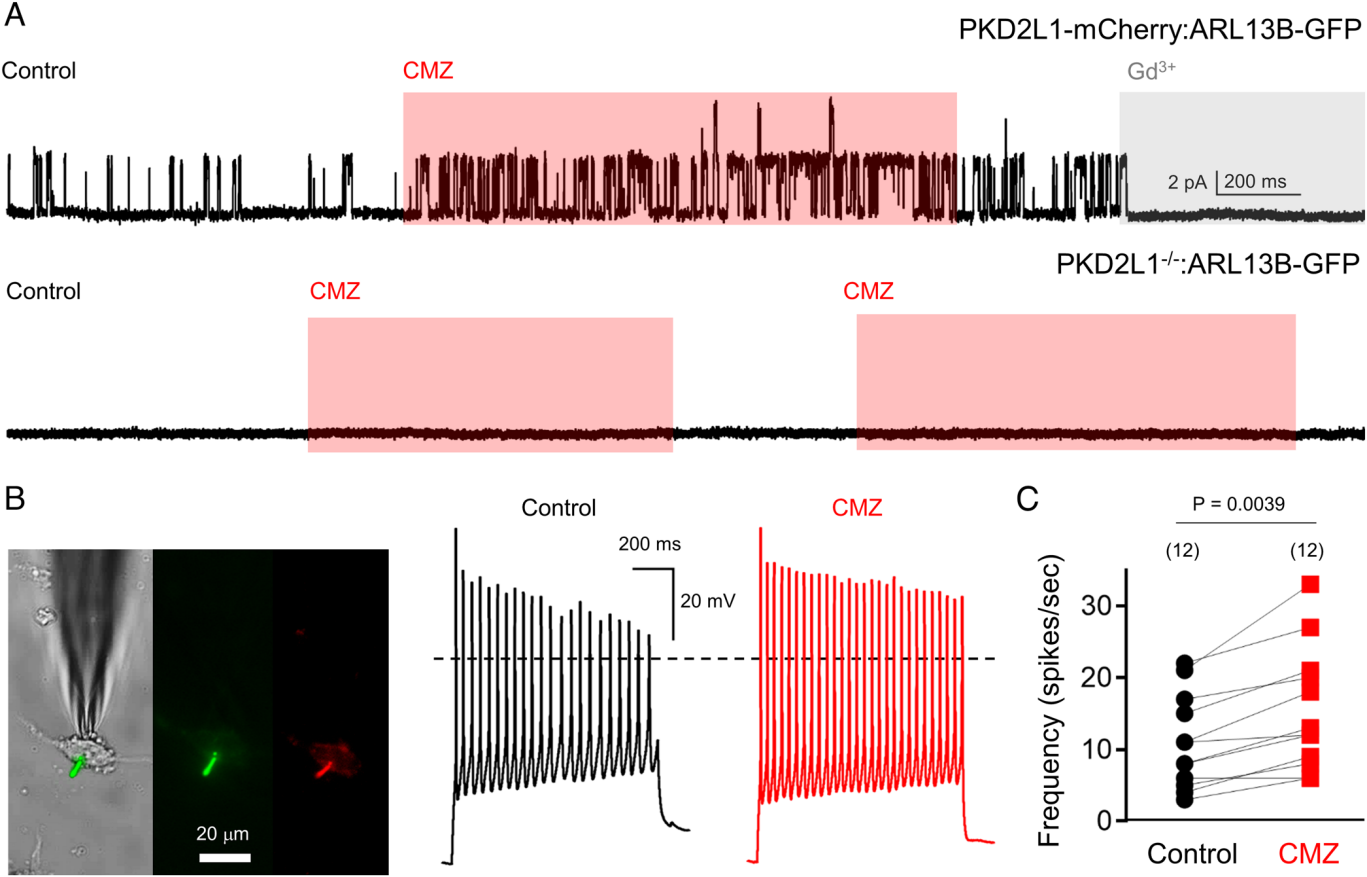
Pharmacological activation of neuronal PKD2L1 increases the frequency of action potential firing. (*A*) *Top*, stimulation and inhibition of ciliary single-channel openings by CMZ and gadolinium (Gd^3+^), respectively, in neurons isolated from PKD2L1-mCherry:ARL13B-EGFP mice. *Bottom*, lack of single-channel events in control and CMZ conditions in an immature ciliary patch recorded from PKD2L1^−/−^:ARL13B-EGFP neurons. Ciliary membranes were held at 40 mV in the on-cilia configuration. (*B*) *Left*, images of current-clamped neurons isolated from PKD2L1-mCherry:ARL13B-EGFP mice after 4 to 8 d in culture using DIC, GFP, and RFP filters. *Right*, example hippocampal action potentials activated by 120 pA current injection before (control) and after 1 µM CMZ bath application. (*C*) Paired scatter plots comparing the maximum number of action potential spikes generated from control and drug treatment conditions. *P*-value results from a two-tailed Wilcoxon matched-pairs rank test are indicated, and the number of neurons recorded from each condition are indicated within parentheses.

While the general expression of PKD2L1 in the brain has been reported, the channel’s subcellular localization and its conductive properties within hippocampal neurons are unknown (34, 35). To address this, we created a transgenic mouse expressing the PKD2L1 gene C-terminally fused with mCherry using the CRIPSR/Cas9 gene–editing method (38). The PKD2L1-mCherry strain was then backcrossed with the ARL13B-EGFP transgenic animals (28). The resulting hybrid, PKD2L1-mCherry:ARL13B-EGFP, provides simultaneous primary ciliary labeling and PKD2L1 localization within living tissues (Movie S2). Both in cultured neonatal hippocampal neurons (P0, P1) and from fixed adult brain slices, PKD2L1-mCherry clearly localized to the primary cilia labeled with ARL13B-EGFP in the CA1–CA3 and DG (Fig. 4*A* and *SI Appendix*, Fig. S5 *A–E*). To determine the endogenous conductive properties of PKD2L1, we used electrophysiology of cultured hippocampal neurons in the “on-cilia” configuration. When Na^+^ was used as a charge carrier, a large inward single-channel conductance (γ = 147 pS) was observed (Fig. 4 *B* and *C*). When Ca^2+^ was used as a charge carrier, the inward ciliary conductance was smaller (γ = 41 pS). However, the neuronal ciliary conductance was more selective for Ca^2+^ than that for Na^+^, based on the 40 mV shift in reversal potential (ψ). Importantly, both conductances are absent from immature ciliary patches (N = 15) recorded from cultured neurons isolated from PKD2L1^−/−^ mice (Fig. 4*B*). The neuronal ciliary channel open probability increases at higher membrane potentials, with a half activation voltage (V_1/2_) equal to 47 ± 7 mV and is within the range of thresholds previously reported for PKD2L1 untagged under heterologous conditions (Fig. 4*D*) (20, 21, 39). The voltage dependence of neuronal ciliary channels harvested from ARL13B-EGFP recordings was similar to PKD2L1-mCherry:ARL13B-EGFP neurons (V_1/2_ = 52 ± 9 mV), indicating that the genetically encoded mCherry tag did not interfere with PKD2L1 gating properties (Fig. 4*D*). Ciliary channel open time was enhanced by 1 µM of the PKD2L1 activator calmidazolium (CMZ) and blocked by 10 µM of the nonselective TRP channel antagonist gadolinium (Fig. 5*A*) (28). CMZ was inert in PKD2L1^−/−^ immature ciliary patch clamp recordings (N = 7). Results from our previous current clamp recordings suggest that PKD2L1 contributes to high-frequency spiking. Consistent with this interpretation, we compared action potentials from neurons before and after CMZ treatment and observed a significant increase (51 ± 11%; average, SD) in the maximum spike frequency (Fig. 5 *B* and *C*).

Since PKD2L1 produces disproportionately large tail currents upon repolarization, we hypothesize that PKD2L1 may contribute to high-frequency spiking by conducting Na^+^/Ca^2+^ ions when the neuronal membrane rapidly repolarizes during the down stroke of the action potential. To assess this, we recorded hippocampal ciliary currents triggered by variable depolarization times and observed enhanced inward single-channel open events upon repolarization (*SI Appendix*, Fig. S6*A*). Upon repolarizing the membrane to −80 mV, normalized integrated currents measured from the neuronal cilium and from HEK cells expressing PKD2L1 had similar activation dependence on depolarization time (*SI Appendix*, Fig. S6*B*). These data suggest that exogenously expressed PKD2L1 channels recapitulate the kinetic and conductance properties of endogenous neuronal PKD2L1 recorded from the primary ciliary membrane. This comparison suggests that ciliary PKD2L1 channels can generate an excitatory inward current upon repolarization, which we propose regulates hippocampal excitability and neurophenotypic behaviors.

## Discussion

Duncan and Dahl first reported primary cilia in the CNS more than 60 y ago (1, 2). While primary ciliary signaling (e.g., hedgehog) in neural tube formation and in the developing brain is well studied, our understanding of ciliary regulation in the adult CNS is limited (40–42). We report that maturation of primary cilia in the hippocampus and cortex requires the expression of PKD2L1 channels. While several other TRP members form excitatory channels in the soma and dendritic compartments of cerebellar, striatal, and hippocampus neurons, their loss of function is not associated with alterations in ciliogenesis and maturation (43). Thus, the primary ciliary phenotype we observe in the CNS is likely due to the specific loss of PKD2L1 expression and not a general feature of impaired neuronal excitability. Importantly, primary ciliary maturation was not impacted in nonneuronal lineages which are commonly impacted in ciliopathy diseases, such as the principal cells of kidney collecting duct in ADPKD (17). Within the greater context of the scientific literature, our findings indicate that specific polycystins function as ciliary calcium channels of divergent tissues, with PKD2L1 occupying the ciliary membrane of hippocampal neurons. Using transgenic ciliary and channel reporter mouse strains, we determine that PKD2L1 localizes to the primary cilia of hippocampal neurons and functions as a large conductance calcium channel. While we cannot rule out PKD2L1 assembling with other channel subunits to form a heteromeric channel complex, the neuronal ciliary conductance magnitude and ion selectivity is most similar to the reported values of homomeric PKD2L1 channels recorded in heterologous expression systems (15, 20, 39).

Neuronal action potentials from PKD2L1 knockout animals are unable to maintain rapid action potential firing under high-current injection. We propose that this is a consequence of losing the TRP channel housed within the ciliary compartment. PKD2L1 is voltage dependent, opening at positive membrane potentials and generating large inward cation currents upon membrane repolarization (20, 21). These are unique characteristics for a TRP channel, and we propose that PKD2L1 contributes to high-frequency action potential spiking by generating a depolarizing (Na^+^, Ca^2+^) conductance when neurons repolarize. Here, PKD2L1’s role in the hippocampus might be a spike generator as proposed in inhibitory cerebrospinal fluid-contacting neurons (CSF-cNs) of the murine brainstem (44, 45). Since we find that the primary cilia of hippocampal neurons extend from the soma, PKD2L1 may also support action potential backpropagation from the axon to dendritic compartments (46). Experiments conducted in fibroblasts indicate cilia are considerably electrically insulated from the cell body (28, 32). However, the electrical coupling between the primary ciliary compartment and soma of excitable neurons is unknown. Future work should establish the junction resistance between the cilium and the soma, and the physiological role of ciliary PKD2L1 channels in neurons. Recently, synapses between axons and primary cilia (i.e., axo-ciliary synapses) are reported to mediate serotonergic signaling via 5-HTR6 GPCR activation in the murine hippocampus (6). The privileged enrichment of PKD2L1 channels and 5-HTR6 receptors within the primary ciliary compartment presents the possibility that serotonin neurotransmitters may modulate channel function and neuronal excitability via downstream GPCR effectors.

Loss of PKD2L1 expression causes increased seizure susceptibility and ASD-like features in mice. How does the loss of this TRP channel—which conducts a depolarizing Ca^2+^ current—result in this apparent hyperexcitable neurophenotype? Excitatory and inhibitory circuit imbalances are defining features of ASD and forms of epilepsy (47). Our results indicate that loss of PKD2L1 expression primarily affects excitability of inhibitory neurons. Thus, circuit disinhibition caused by impaired excitability of inhibitory neurons may underlie the hyperexcitable phenotype observed in these mice. Although there are no reports associating human PKD2L1 variants with ASD or epilepsy, the neurophenotypic combination observed in PKD2L1 knockout mice reflects comorbidities presented in patient populations. Epilepsy coassociates with ASD in approximately one-third of affected individuals (48–50). Approximately 27% of the impacted genes associated with epilepsy are caused by ion channel variants, and several other CNS channel (e.g., Na_v_1.2) variants are associated with ASD (51–53). Thus, while the direct genetic association of PKD2L1 with human neurodevelopmental diseases has not been reported, there is considerable precedent for loss of ion channel function altering neuron excitability and precipitating in epilepsy–ASD conditions.

## Methods

### Isolation of Primary Hippocampal Neurons.

Hippocampi were dissected from three to six P0-P1 mice and digested in ~20 units of papain (LK003176, Worthington) and ~200 units of deoxyribonuclease (LK003172, Worthington) dissolved to basal medium eagle solution (B1522, Sigma) at 37 °C for 25 min. Tissues were washed with BME and triturated to release cells. Cells were centrifuged at 300 × g for 4 min and plated on polylysine-coated glass coverslip at 3.5 × 10^5^ density in basal medium eagle solution containing B27 supplement (17504044, Gibco), N-2 supplement (17502048, Gibco), 0.5% penicillin/streptomycin (15140148, Gibco), 5% fetal bovine serum, 5% horse serum (260500, Gibco), and GlutaMax (350500, Gibco). The media was replaced after 5 h with the same solution containing 0% serum and 2.5 µM cytosine β-D-arabinofuranoside hydrochloride (C6645, Sigma). Hippocampal neurons were cultured for 4 to 14 d prior to conducting imaging and electrophysiology experiments.

### Electrophysiology.

Soma current clamp recordings were performed at room temperature with MultiClamp 700B (Molecular Devices) connected to Digidata 1550 digitizer. Current and membrane potential traces were sampled at 20 kHz and filtered at 4 to 10 kHz. The patch pipettes were pulled from borosilicate glass capillary tubes (WPI) using a P-87 micropipette puller (Sutter Instruments). The pipette resistance was ~2 to 5 MΩ when the patch pipette was filled with intracellular solution. Neuronal membrane potential was measured in whole-cell configuration with intracellular solution containing (in mM): 125 K-gluconate, 10 KCl, 1 EGTA, 0.1 CaCl_2_, 10 NaCl, 10 HEPES, 5 tris-phosphocreatine, 4 MgATP, 0.5 NaGTP, pH adjusted to 7.3 with KOH, and osmolarity raised to 305 mOsm/L with mannitol. Two to three mg/mL biocytin (B4261, Sigma) was added and sonicated on the day of the experiment. The external solution contains (in mM): 140 NaCl, 5.3 KCl, 1.8 CaCl_2_, 1 MgCl_2_, 10 HEPES, 10 glucose, pH adjusted to 7.4 with NaOH, and osmolarity raised to 305 mOsm/L with mannitol. To label recorded neurons, 2 to 3 mg/mL biocytin (B4261, Sigma) was added to the pipette solution on the day of the experiment. Membrane capacitance and series resistance were compensated. Hippocampal cultures were recorded at day in vitro 4 to 16.

Single-channel currents were measured from the primary ciliary membrane an Axon 200B (Molecular Devices) amplifier connected to Digidata 1550 digitizer, as previously described (17, 28, 30). The standard pipette solution contained (in mM): 100 NaCl, 10 HEPES, and pH7.4 with NaOH and adjusted to 300 to 305 mOsm/L with mannitol. High-potassium bath solutions containing 130 KCl, 15 NaCl, 10 HEPES, 1.8 CaCl_2_, 1 MgCl_2_, pH7.4 with KOH, and 300 to 305 mOsm/L were used to neutralize the membrane potential. To measure the calcium conductance of the neuronal ciliary membrane, the pipette solution contained (in mM): 100 CaCl_2_, 10 HEPES, and pH 7.4 with CaOH and adjusted to 300 to 305 mOsm/L with mannitol. Drug stocks of 10 mM CMZ (C3930, Sigma) were formulated in dimethyl sulfoxide (D2650, Sigma) and kept at −20 °C until the day of use.

### Immunocytochemistry.

To identify inhibitory and excitatory neurons from the current clamp experiments post hoc, 1 nM Biocytin Alexa Fluor 594 (ThermoFisher) was added to the intracellular solution. The cultured neurons on coverslips were fixed in 4% paraformaldehyde for 20 min after completion of the electrophysiological experiment. They were washed and incubated overnight at 4 °C with PBS solution containing 1% NGS, 0.1% Triton-X, and the following primary antibodies: 1:500 mouse anti-GAD67 (MAB5406, Sigma) and 1:500 rabbit anti-MAP2 (17490-1-AP, Proteintech). After washout of primary antibodies, the following secondary antibodies were added: 1:1,000 goat anti-mouse Alexa Fluor 488 (A32723, Invitrogen), 1:1,000 goat anti-rabbit Alexa Fluor 647 (A21244, Invitrogen), and 1:1,000 streptavidin-Alexa Fluor 568 conjugate (S11226, Invitrogen) for an hour at room temperature. The coverslips were mounted on Prolong Gold (P36930, Invitrogen) and dried for 24 h. Images were obtained using an inverted 100× oil immersion objective (1.45 numerical aperture) on a Nikon A1 confocal microscope. To visualize primary ciliary and PKD2L1-mCherry localization in situ, animals were perfused with 4% paraformaldehyde and tissue was isolated and immersed for 72 h in 30% sucrose. The brain and other organs were serially cut into 20 µm sections using a Leica microtome. For immunohistochemistry, the brain sections were rinsed in PBS and incubated for 4 h at room temperature in primary antibody. Following PBS rinses, the tissue was incubated for 2 h at room temperature in secondary antibody. The slides were rinsed in PBS, mounted onto Superfrost microscope slides, and coverslipped with Prolong Gold antifade reagent. The images were obtained using the Nikon A1 confocal microscope.

### Transgenic Animals and Behavioral Tests.

PKD2L1^−/−^ knockout mice (B6.Cg-*Pkd2l1^tm1.1Yuni/J^*) were obtained from Jackson Laboratory and were crossed with our previously developed ARL13B-EGFP transgene mice (28, 33). The resulting PKD2L1 heterozygous (PKD2L1^+/−^:ARL13B-EGFP) and homozygous (PKD2L1^−/−^:ARL13B-EGFP) hybrids were genotyped using the primers previously described (32, 54). PKD2L1-mCherry C57BL/B6 mice were generated using the CRISPR/Cas9 method (38, 55) to insert an mCherry tag on the C terminus of the PKD2L1 gene (ENSMUST00000042026.5). Three CRISPR guild oligonucleotides were used to generate the founder strains: GAACCTGTATAATCCGTCCT, AGCTGGAGTGACACCTAGGA, and AGGGAGCTGGAGTGACACCT. The founders bred with the C57BL/B6 mice produced an N1 generation and were backcrossed with the ARL13B-EGFP strain (28). The mice were genotyped using the following primers: Pkd2l1_SeqF: GCCTTCTAGTTGCCAGCCAT; Pkd2l1_SeqR: TCACCTTCAGCTTGGCGG. The marble-burying test was administered using the following procedure. Experimenters were blinded to the genotype of the mice. For each test mouse, one standard housing cage was filled to a depth of 5 cm with clean wood chip bedding. Fifteen clean marbles were placed on top of the bedding, arranged in three columns and five rows. For testing, one mouse was placed in each cage and left undisturbed for 30 min. A photograph of each test cage was taken to record the number of buried marbles. Marbles that were buried at least 2/3 of its depth were considered buried. Open-field tests were administered as follows. The mice were placed in the center of a 56 × 56 cm arena, and their movement was digitally monitored for 5 min (Limelight software, Actimetrics). The mice were tracked to determine the total distance moved and percent of time in the center (20 cm × 20 cm). Zero-maze tests were conducted on an elevated round track with a 56-cm diameter. The track was divided into four equal quarters. One quarter had walls (closed arena) followed by another quarter without walls (open arena) and this pattern was repeated for the whole track. The test mouse was placed into the open arena and allowed to explore for 5 min. Movement of the mouse was digitally tracked using LimeLight software (Actimetrics) to determine the amount of time it spent in the open arena as a percentage of the total time. Y maze tests were conducted in a Y-shaped arena with three arms of equal length (3.2 cm at the base and 13.5 cm at the top; side walls were 18 cm wide). The test mouse was placed at the base of the Y, with its nose pointed toward the center of the arena. The mouse was digitally monitored with LimeLight software (Actimetrics) for 5 min, and the order of arm entries was analyzed for spontaneous alternation (SA). A spontaneous alternation is defined as three consecutive choices without repeated entries into the previous arm explored. An alternation is scored using the following formula: # alternations/# of possible alternations * 100. Three-chamber social interaction testing was performed in a rectangular, three-chambered apparatus of equally sized chambers. The mice were first habituated to the center chamber for 10 min followed by a 10-min habituation to all the three empty chambers. The test mice were removed temporarily and an upside-down wire pencil cup with a weight was placed on the left chamber and a novel mouse was placed inside an upside-down wire pencil cup with a weight on the top in the right chamber. After both wire cups were in place, the test mouse was placed in the middle chamber and the time spent sniffing the novel mouse or the novel object was recorded for 10 min using LimeLight software (Actimetrics). After each test session, the apparatus was cleaned with 70% ethanol.

### Electroencephalogram (EEG) Recordings and Seizure Induction.

Ten-to-twelve-week-old male littermates were used for EEG recordings in awake, ambulatory mice. The week before an EEG recording, deeply anesthetized mice were implanted with prefabricated EEG/EMG head mounts (Pinnacle Technology) 2.0 mm posterior to the bregma, along the midsagittal suture, and allowed to recover for 1 wk. The mice were then monitored for EEG activity using Sirena software (Pinnacle Technology). Baseline activity was monitored for 30 min followed by an i.p. injection of 25 mg kg^−1^ kainic acid (Tocris). Following the kainic acid injection, the mice were monitored to determine the amount of time spent seizing during the 120-min trial, the onset of SE, and the latency to the first epileptiform activity. Epileptiform activity was defined as events lasting greater than 10 s with amplitudes at least twice the SD of the baseline and separated from another event by at least 5 s. SE was characterized as prolonged epileptiform activity longer than 5 min in duration with no silent period greater than 10 s. EEGs were quantified using LabChart v8 (ADInstruments).

### Statistics.

Unless specified otherwise, statistical analyses were performed using Student’s *t* test (unpaired, two tailed) or ANOVA using Prism software (GraphPad). The data bars and error bars indicate mean ± SEM for behavioral tests.

## Supplementary Material

Appendix 01 (PDF)

Movie S1. **Three dimensional movie of a hippocampal brain section (1.1 mm: 1.1 mm: 10 μm) harvested from an ARL13B-EGFP mouse.** Images were acquired from a fixed brain section on a confocal microscope and imaged at 10 mm of depth. The ARL13B-EGFP signal is enriched in the primary cilia, red signal indicates the γ- tubulin (centrosome) antibody localization at ciliary base and blue DAPI staining indicates the nuclei.

Movie S2. **Three dimensional movie of the CA1 region (110 μm: 110 μm: 10 μm) harvested from an PKD2L1-mCherry:ARL13B-EGFP mouse.** As in Movie S1, confocal images were acquired from a fixed brain section on a confocal microscope and imaged at 10 μm of depth. Note the PKD2L1-mCherry (red) signal is enriched in the primary cilia illuminated by the ARL13B-EGFP (green) signal and DAPI staining (Blue) indicates the nuclei

## Data Availability

Source data have been deposited in Northwestern University institutional repository service (https://arch.library.northwestern.edu/concern/datasets/s4655h05q) (56).
