# Supplementary material for: Primary cilia TRP channel regulates hippocampal excitability
Source: Proc Natl Acad Sci U S A. 2023 May 22;120(22):e2219686120. doi: 10.1073/pnas.2219686120 (PMC10235993; doi:10.1073/pnas.2219686120)
Supplement: Supplementary file 1 — Appendix 01 (PDF) [file pnas.2219686120.sapp.pdf]

PNAS

Supporting Information for

Primary cilia TRP channel regulates hippocampal excitability.

Thuy N. Vien[1*], My C. Ta[1*], Louise F. Kimura[1], Tuncer Onay[2] and Paul G. DeCaen[1**]

1) Department of Pharmacology, Feinberg School of Medicine, Northwestern University, 320 East Superior, Chicago, Illinois 60611, USA.
2) Center for Genetic Medicine; Feinberg School of Medicine, Northwestern University, 320 East Superior, Chicago, Illinois 60911, USA

* These authors contributed equally to this work.
**Corresponding author.

Paul G. DeCaen PhD
Email: paul.decaen@northwestern.edu

This PDF file includes:

Figures S1 to S6
Legends for Movies S1 to S2

Other supporting materials for this manuscript include the following:

Movies S1 to S2

Supplementary Figures

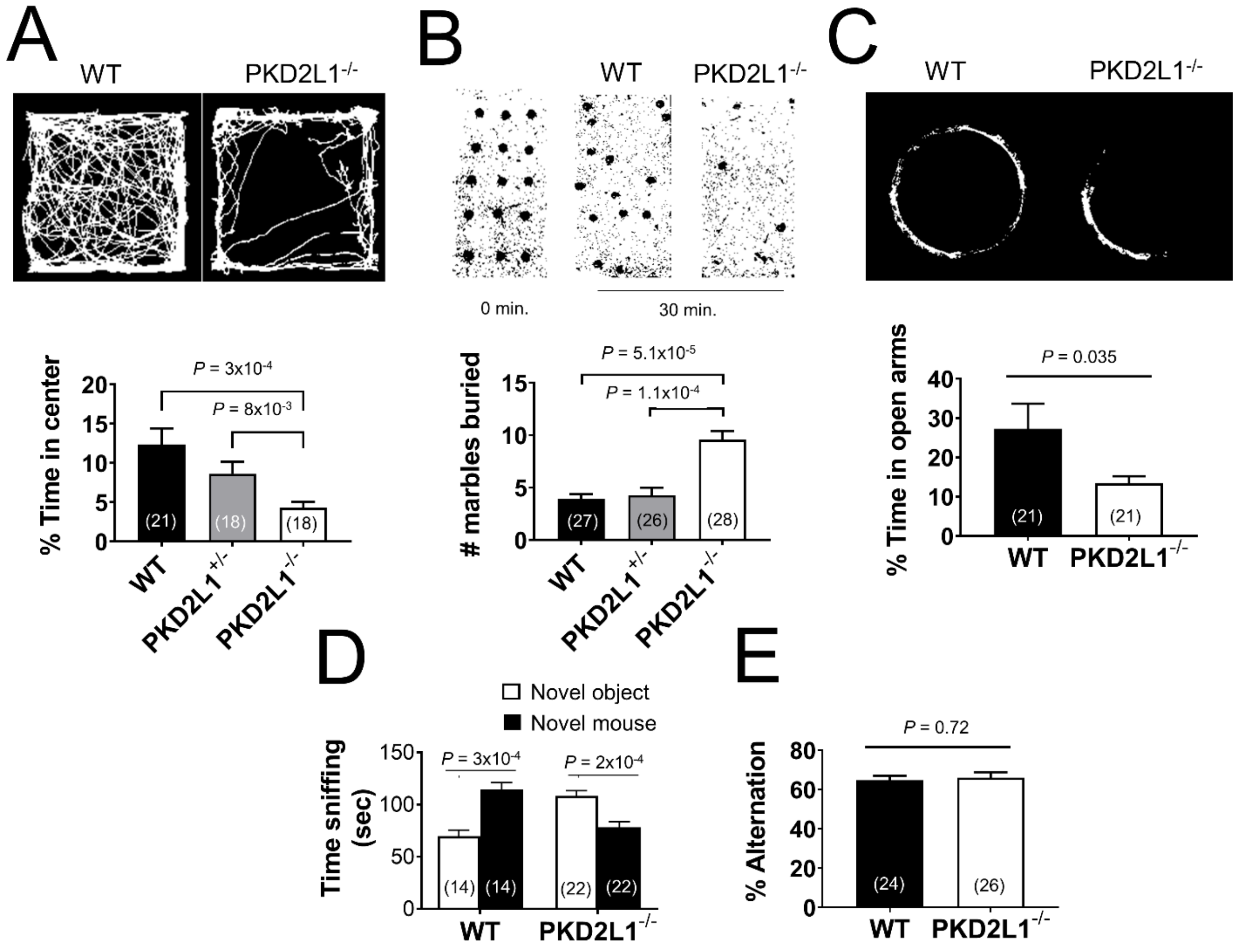

Fig S1. PKD2L1 knockout mice exhibit ASD-like behaviors.

A) Results comparing WT and PKD2L1$^{-/-}$ mice using the open field behavioral test. *Top*, lines plotting locomotor trajectories during the 5 minute test period. *Bottom*, bar graphs comparing total distance and percent time spent at the center of the cage as readouts for locomotor activity and anxiety, respectively. **B**) Results comparing mouse genotypes using the marble burying assay designed to assess repetitive-like behaviors. The number of marbles buried in 30 min of testing is measured across the different genotypes. **C**) Results from the elevated zero maze assay to measure anxiety-like behavior. The percentage of time spent in the open arms of the maze were measured during 5 min of testing. **D**) Results from the three-chamber social interaction test used to assay sociability in the mice. In a three- chambered box, the amount of time mice spent sniffing a novel mouse compared to a novel object was monitored and the total amount of time was quantified for each subject mouse during 10 min trials. **E**) There were no differences in spontaneous alternation between WT and PKD2L1$^{-/-}$ mice in the Y maze spontaneous alternation test, a commonly used assay to assess short term spatial working memory. Sample sizes are indicated in the parenthesis.

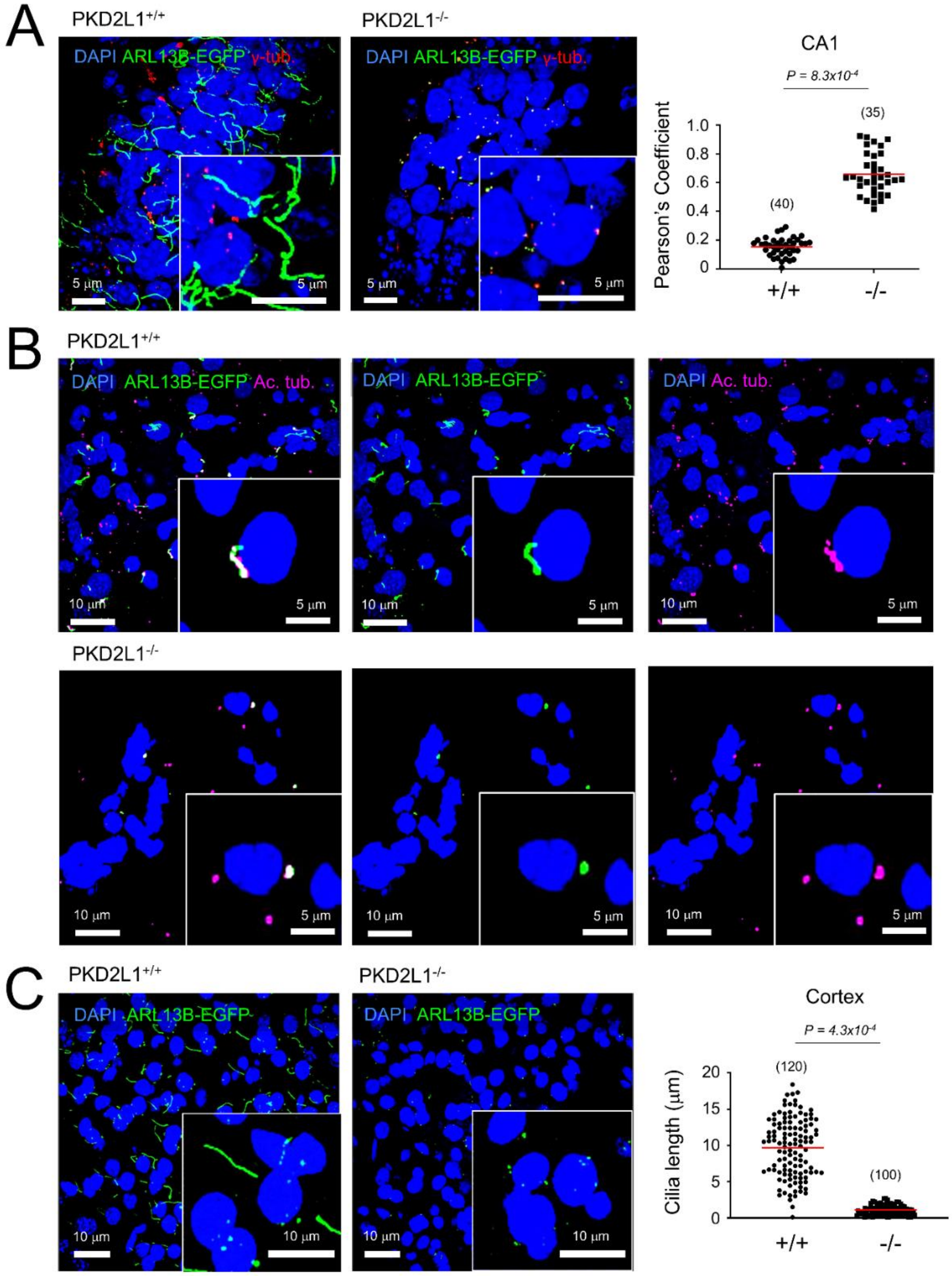

Fig S2. Confirming of loss of neuronal primary cilia from PKD2L1$^{-/-}$ hippocampus and cortex.

A) Confocal images of CA1 brain sections from PKD2L1$^{+/+}$:ARL13B-EGFP and PKD2L1$^{-/-}$:ARL13B-EGFP mice treated with DAPI (nucleus) and immunolabeled for the centrosome component, γ-tubulin. *Right*, Pearson's coefficient analysis of colocalization of ARL13B and γ-tubulin. Number of images analyzed are indicated within the parenthesis. Average coefficient is indicated by red lines. **B**) Confocal images of CA1 brain sections from PKD2L1$^{+/+}$:ARL13B-EGFP and PKD2L1$^{-/-}$:ARL13B-EGFP mice treated with DAPI (nucleus) and immunolabeled for acetylated-tubulin (primary cilia), confirming the loss of primary cilia from knockout animals. **C**) Loss of PKD2L1 expression results in immature primary cilia in the cortex. *Left*, example confocal images of cortex brain sections from PKD2L1$^{+/+}$:ARL13B-EGFP and PKD2L1$^{-/-}$:ARL13B-EGFP mice treated with 4',6-diamidino-2-phenylindole (DAPI). *Right*, scatter plots of primary cilia length. Number of images analyzed are indicated within the parenthesis. Average length is indicated by red lines.

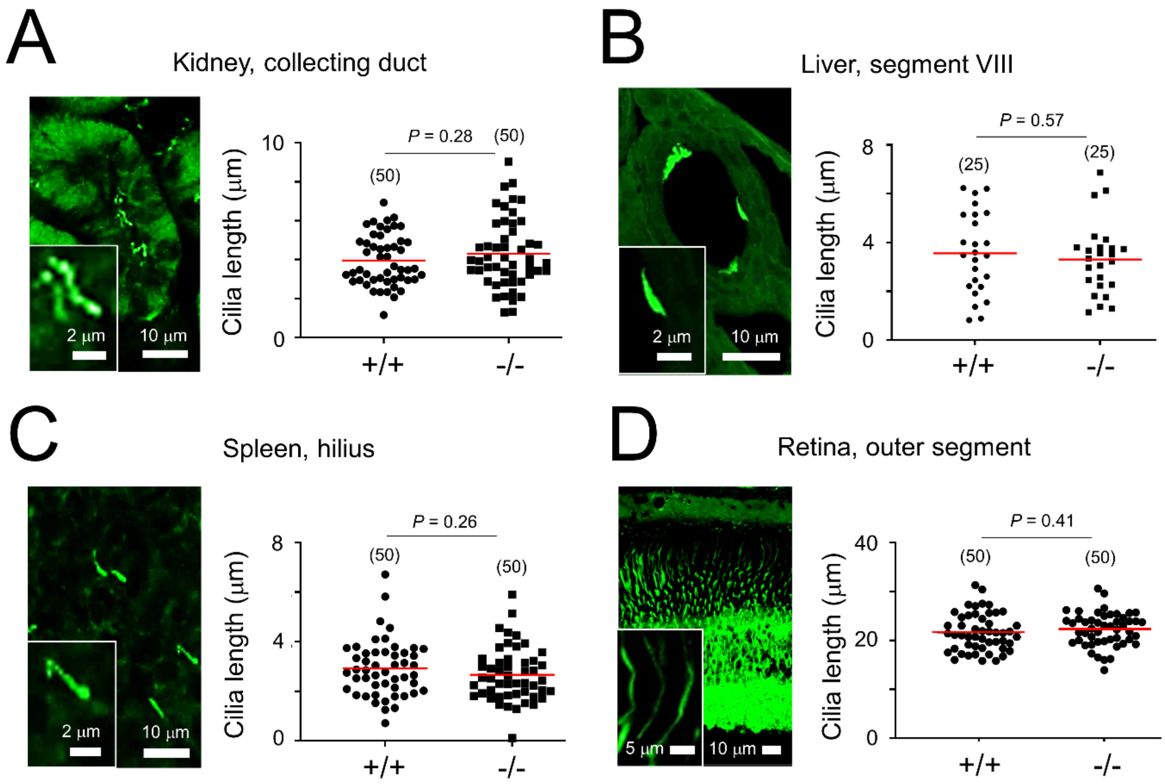

Fig S3. Loss of PKD2L1 does not impair primary cilia maturation in non-neuronal tissues.

A-D) Example confocal images and respective cilia length analysis from tissues commonly impacted by ciliopathy disease. All example images are from tissues harvested from PKD2L1$^{+/+}$:ARL13B-EGFP mice. P-values resulting from Student's t-tests are indicated above each graph. Average length is indicated by red lines. Number of cilia imaged in each data set is indicated in the parenthesis.

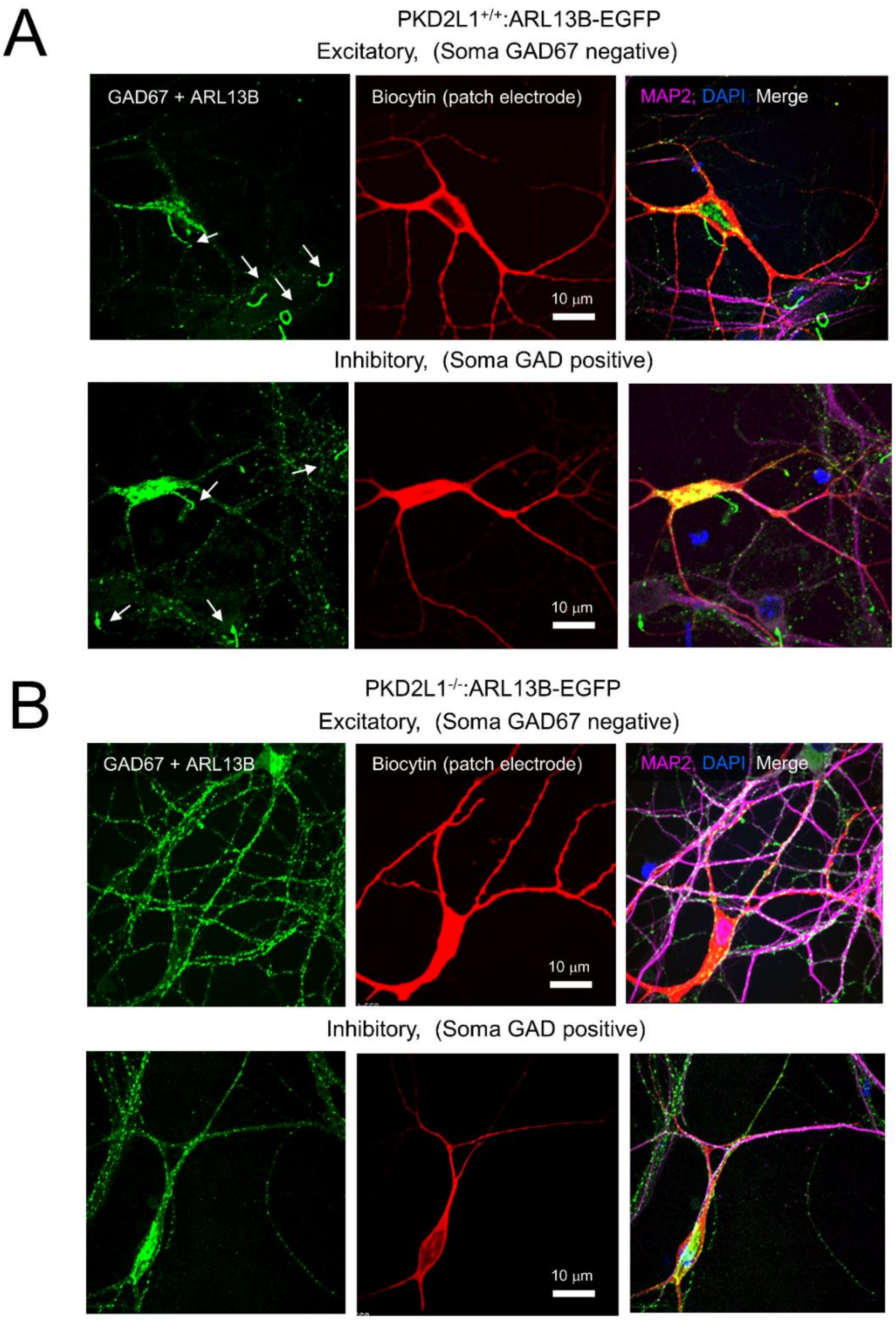

Fig. S4. Identification of excitatory and inhibitory neurons after current clamp experiments.

A, **B**) Example confocal images of fixed cultured neurons used to identify inhibitory/GABAergic interneurons for the analysis in Figure 3. Patch electrodes were loaded with biocytin to identify the current clamped neuron under the red emission. Green fluorescence identifies the transgenic ARL13B-EGFP protein in the primary cilia (arrows) and the immunofluorescence labeled glutamic acid decarboxylase 67 (GAD67), which is highly expressed in the soma (asterisks) of inhibitory neurons. Merged confocal images of neurons include nuclei labeled with DAPI or microtubule associated protein 2 (MAP2).

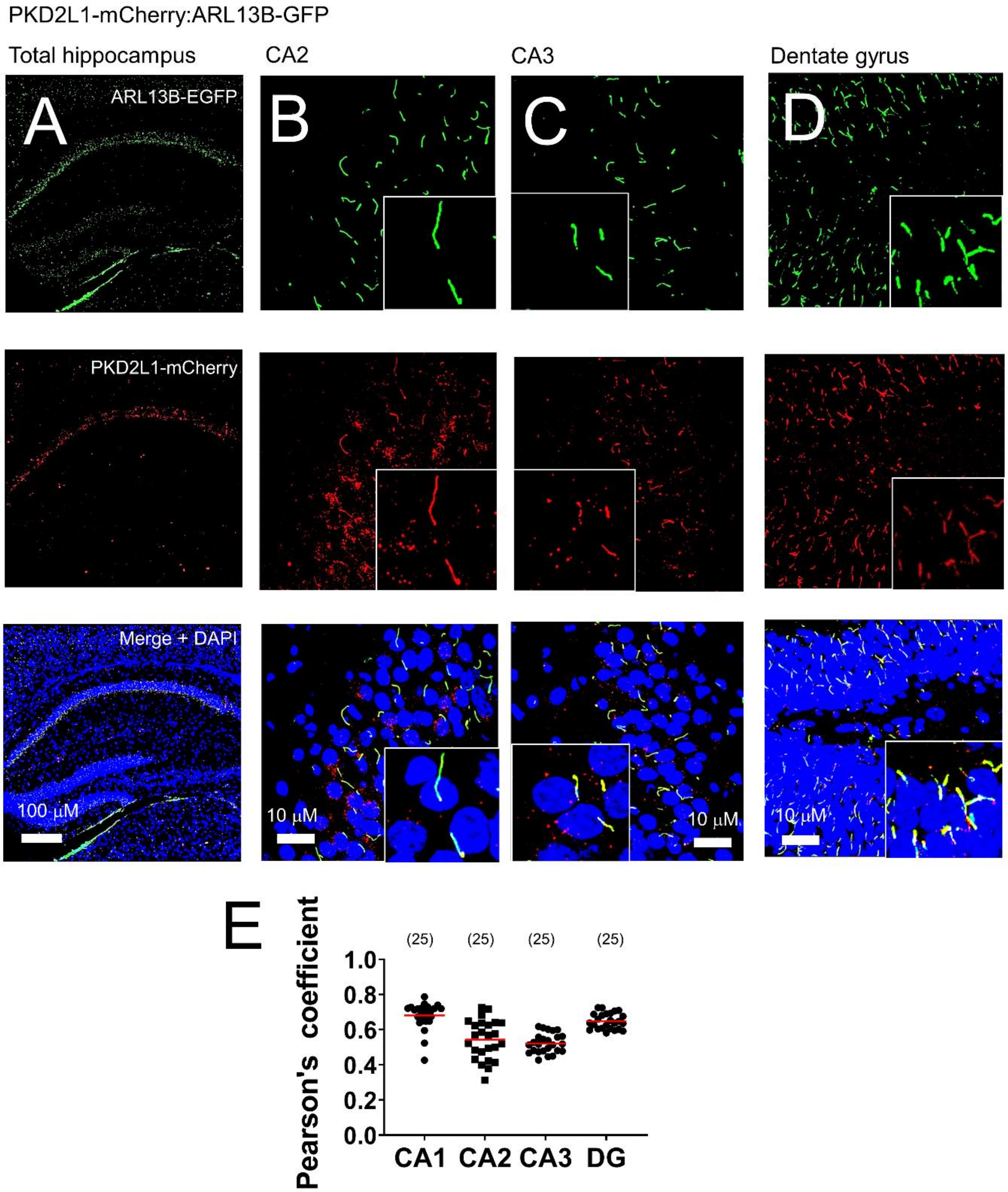

Fig S5. PKD2L1-mCherry localizes to neuronal primary cilia in intact hippocampal brain slices.

A) Global stitched confocal images of a fixed hippocampal brain slice harvested from a 3 month old PKD2L1-mCherry:ARL13B-EGFP mouse visualizing primary cilia (transgene ARL13B-EGFP, green), nuclei (DAPI staining, blue) and location of mCherry labeled channels (Red). **B-D)** Confocal images of the CA2, CA3 and dentate gyrus regions of the hippocampus. *Inset*, expanded views of mature and immature primary cilia taken from PKD2L1$^{+/+}$ or PKD2L1$^{-/-}$ mice. *Right*, corresponding analysis of the mCherry channel colocalization with the EGFP ciliary signal. **E**) Pearson's coefficient analysis of colocalization of PKD2L1-mCherry with the ciliary ARL13B-EGFP fluorescence. Horizontal red line indicated the average coefficient and sample size was 25 cilia imaged per brain location

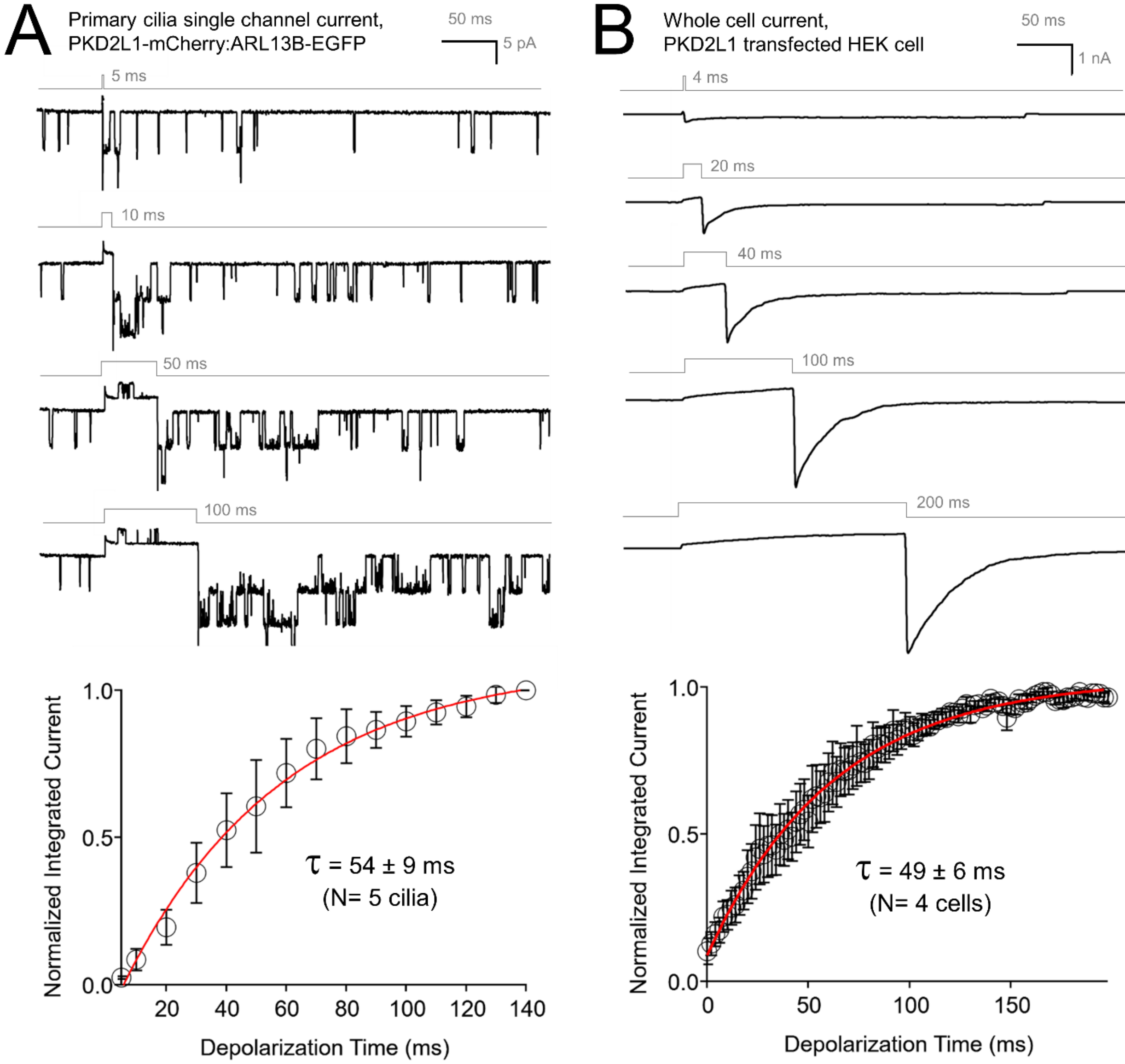

Fig S6. PKD2L1 contributes to macroscopic tail currents in the hippocampal neuron primary cilia.

A) *Top*, exemplar inward open channel events (black) triggered by membrane repolarization (-80 mV) after variable 30 mV depolarization times (gray). *Bottom*, depolarization time dependence of integrated single channel current activation. Inward single channel currents were integrated during the 300 ms phase after repolarization and normalized for each cilia recording. The resulting relationship is fit to a single exponential equation (red). **B**) *Top*, exemplar PKD2L1 whole cell currents activated by membrane repolarization (-80 mV) after variable 30 mV depolarization times (gray). *Bottom*, analogous to the analysis performed in B, normalized integrated inward tail currents were fit to a single exponential equation (red).

Movie S1 (separate file). Three dimensional movie of a hippocampal brain section (1.1 mm: 1.1 mm: 10 μm) harvested from an ARL13B-EGFP mouse.

Images were acquired from a fixed brain section on a confocal microscope and imaged at 10 mm of depth. The ARL13B-EGFP signal is enriched in the primary cilia, red signal indicates the γ- tubulin (centrosome) antibody localization at ciliary base and blue DAPI staining indicates the nuclei.

Movie S2 (separate file). Three dimensional movie of the CA1 region (110 μm: 110 μm: 10 μm) harvested from an PKD2L1-mCherry:ARL13B-EGFP mouse.

As in Movie S1, confocal images were acquired from a fixed brain section on a confocal microscope and imaged at 10 μm of depth. Note the PKD2L1-mCherry (red) signal is enriched in the primary cilia illuminated by the ARL13B-EGFP (green) signal and DAPI staining (Blue) indicates the nuclei
